# Implementation of an e-Tool (the Provider Asthma Assessment Form) Integrated Into the Electronic Medical Record in Primary Care: Mixed Methods Survey of Perceived Utility, Practitioner Satisfaction, Barriers and Enablers

**DOI:** 10.2196/80399

**Published:** 2026-06-03

**Authors:** Matheson L McFarlane, Alison Morra, Delanya Podgers, David Barber, Kelsey Ruetz, M Diane Lougheed

**Affiliations:** 1 Asthma Research Unit, Kingston Health Sciences Centre, 76 Stuart Street, Kingston, ON, K7L 2V7, Canada, 1 6474608012; 2 Division of Respirology, Department of Medicine, Queen's University, Kingston, ON, Canada; 3 Kingston Health Sciences Centre, Kingston, ON, Canada; 4 Canadian Primary Care Sentinel Surveillance Network, Kingston, ON, Canada; 5 Department of Family Medicine, Queen's University, Kingston, ON, Canada

**Keywords:** asthma, severe asthma, diagnosis, pulmonary function tests, guideline implementation, digital tools, electronic forms, best practice, knowledge translation

## Abstract

**Background:**

Asthma care gaps between best practice and clinical practice contribute to the burden of asthma on individuals and society. Electronic medical records (EMRs) provide a unique opportunity to integrate novel e-tools at the point of care.

**Objective:**

The purpose of this study was to evaluate the perceived utility, health care practitioner satisfaction, and barriers and enablers associated with the implementation of the Provider Asthma Assessment Form (PAAF), a novel e-tool integrated into the primary care EMR.

**Methods:**

Health care practitioners (n=80) at a family health team were invited by email to participate in a voluntary survey regarding their use of the PAAF in their role within the family health team. Respondents who had used the PAAF were asked to assess its perceived utility, their satisfaction with it, and any associated barriers and enablers. Responses were analyzed using descriptive quantitative analysis and qualitative analysis to identify major themes.

**Results:**

In total, 18 responses were included, including 4 (22.2%) from practitioners who had used the form and 12 (66.7%) from practitioners who had not. Overall, most practitioners who used the form were satisfied with the PAAF and reported that it was helpful in clinical practice, aided decision-making, and was user-friendly. Enablers such as detailed documentation, decision support, and multidisciplinary involvement were identified. Several barriers were also identified, including time constraints, lack of knowledge and training regarding the use of the form, limited opportunity to use it, and limited availability of necessary data elements to complete the PAAF.

**Conclusions:**

The PAAF was perceived to be a useful and largely satisfactory e-tool by responding practitioners. However, several barriers limited user uptake and sustained use. Future directions for PAAF implementation include increased tailoring to the needs of primary care teams and leveraging technological advancements. Lessons learned from the PAAF can inform the development and implementation of novel e-tools in primary care EMRs.

## Introduction

Integrating clinical guidelines into primary care asthma management practices remains a challenge globally. It is recognized within the literature that care gaps between best practice and current primary care practice contribute to the burden of asthma on individuals and society [1-7]. As recognized by the Canadian Thoracic Society (CTS), increasing attention is being directed toward developing novel guideline implementation strategies and tools, as guideline dissemination alone has proven insufficient, demonstrated by the persistent asthma care gaps in Canadian primary care practice [8-10]. Hence, knowledge translation (KT) initiatives aimed at reducing asthma care gaps are justified and supported by organizations such as the CTS to assist in the implementation of guidelines across various practice settings [8,11-14]. Research has supported the effectiveness of using implementation frameworks, such as the knowledge-to-action (KTA) cycle, and KT tools to implement asthma clinical guidelines into primary care practice [14-17]. The advent of electronic medical records (EMRs) in primary care provides a unique opportunity to use e-tools and technology as means of KT [18]. The use of e-tools integrated within EMRs expands the possibilities for sentinel surveillance, outcomes monitoring, performance evaluation, and quality improvement in primary care.

Although the implementation of validated e-tools has proven to be valuable for supporting asthma management in primary care, the long-term uptake of e-tools remains low [19-21]. For example, e-tools such as the Breathe app and the electronic Asthma Management System have been integrated within Ontario primary care EMRs and proved to be efficacious tools during evaluation, yet uptake beyond the scope of the research interventions remained low for both tools [16,22]. A study evaluating how to best integrate primary care user preferences into e-tool design revealed themes such as prioritizing the end user, developing for existing workflows (ie, EMRs), ensuring an outcome-oriented design, and gaining stakeholder trust [23]. Therefore, it is imperative that novel primary care asthma management e-tools consider primary care health care practitioners throughout the processes of design, implementation, and continued use.

The Provider Asthma Assessment Form (PAAF) is a novel, point-of-care e-tool designed for use in primary care EMRs. It has an embedded severe asthma algorithm, providing real-time clinical decision support to aid in identifying and managing patients with potentially severe or uncontrolled asthma [24,25]. The PAAF was developed to aid in bridging the gap between best practice and current primary care practice, as it is congruent with current CTS guidelines, includes Pan-Canadian Respiratory Initiative for Electronic Health Records standardized data elements, and incorporates Primary Care–Asthma Performance Indicators [25-27]. Overall, the PAAF is a new e-tool designed to enable quality improvement, performance evaluation, and the implementation of best-practice asthma management in primary care. In a previous study, before formal implementation, the PAAF was found by a focus group of primary care practitioners and key stakeholders to have high value in primary care and to support quality improvement [28]. Within the focus group, 83.3% (n=6) of respondents perceived the PAAF to be a beneficial tool for quality improvement and to have value in primary care [23]. To date, it has been implemented in an EMR of a family health team (FHT) and has been available for 2 years.

The framework used for the development and implementation of the PAAF is the KTA framework [15]. The KTA framework includes aspects of both knowledge creation and knowledge action. The knowledge creation portion is intended to aid in synthesizing evidence and creating KT tools such as e-tools [15]. The knowledge action portion is intended to guide the implementation of KT tools and includes the following steps: identify the problem and select knowledge (ie, guidelines); adapt to the local context; assess barriers and facilitators; select, tailor, and implement interventions; monitor knowledge use; evaluate outcomes; and sustain knowledge use [15]. Hence, using this framework, throughout the process of implementing the PAAF, it is important to understand the barriers and enablers, monitor its use, and develop strategies to sustain the use of the new e-tool.

The impact of the multifaceted intervention of implementing the PAAF in a primary care practice was the focus of a previous study and was associated with improvements in evidence-based recommendations for asthma management and care [17]. However, uptake of the PAAF was low, and care gaps remained. Therefore, the purpose of this study was to assess practitioners’ perspectives on the PAAF following implementation in an FHT setting. In Canadian primary care practice, an FHT involves a team-based, collaborative approach to primary health care delivery, involving a multidisciplinary team of physicians, nurse practitioners, nurses, and allied health care practitioners to provide high-quality care for patients [29]. We aimed to understand the perceived utility, practitioner satisfaction, and barriers and enablers related to the use of the PAAF by primary care practitioners, to gain valuable insight into the implementation of the novel e-tool and identify potential strategies to sustain its use. This research will aid in understanding methods to improve available asthma e-tools, such as the PAAF, and provide valuable insight into future e-tool development that prioritizes the end user.

## Methods

### Ethical Considerations

The study received ethics approval from the Queen’s University Health Sciences and Affiliated Teaching Hospitals’ Research Ethics Board (HSREB #6036789). Informed consent was obtained, and all participants had the opportunity to explicitly opt out of the survey. All data were deidentified for analysis. Identifiable data were securely stored behind the Kingston Health Sciences Centre firewall on a secure drive accessible only to study personnel.

### Study Design

To evaluate the multifaceted implementation of the PAAF (including training sessions for first-year residents, reminder emails, posters, a grand rounds presentation, and summary documents) by primary care practitioners at an FHT in Kingston, Ontario, a questionnaire was created by a second-year family medicine resident. The survey was initially tested following a 1-month introduction period in October 2022 and modified by the study team for the formal postintervention questionnaire distribution. The multifaceted intervention took place from October 2022 to July 2024 (a 20-month period). During the intervention, primary care practitioners were exposed to PAAF reminder documents at the FHT (including reminder emails regarding its availability, summary documents, and reminder posters in clinic team rooms) and had the opportunity to learn about the PAAF during a grand rounds presentation in November 2023. First-year residents received training presentations during their rotations at the FHT to ensure that all practitioners received information and training on the PAAF. The questionnaire was sent by email to all eligible practitioners at the FHT in the last month of the intervention, July 2024. One follow-up email reminder was sent to practitioners.

The survey was developed to assess practitioner satisfaction and perceived utility on a 5-point Likert scale, as well as to assess key barriers to the PAAF’s use following the conclusion of the PAAF implementation study at the FHT. The questionnaire was designed to understand perceived utility, practitioner satisfaction, and barriers and enablers related to the use of the PAAF in primary care practice.

Survey inclusion criteria consisted of family medicine residents, attending physicians, nurse practitioners, registered nurses, nursing students, and other health care workers (ie, clinical pharmacists) providing patient care at the FHT, who were invited to participate by email, for a total of 80 eligible practitioners. Family medicine residents and attending physicians were most likely to have used the form due to the educational initiatives during the intervention; however, all health care practitioners were introduced to the form and received educational content on its availability and applicability in clinical practice. Although students are not consistent members of the FHT, they were invited to participate as the PAAF was used for an asthma community project at the FHT. The questionnaire was provided as a digital link to a Qualtrics XM survey. All survey responses were anonymous, and 18 responses were recorded within the online Qualtrics XM platform.

The survey questions were designed by a family medicine resident and were pretested in October 2022 following a 1-month introductory period for family medicine residents using the PAAF. The survey was subsequently edited with guidance from an attending family physician, respirologist, registered nurse, nurse practitioner, and graduate student. The survey comprised 2 sections, for a total of 11 questions. The study questions included a mix of 5-point Likert scale questions, multiple checkboxes, and open-ended feedback. Overall, the questionnaire was developed to preliminarily assess perceived utility, practitioner satisfaction, and barriers to PAAF use for practitioners who had used the PAAF and to assess the barriers to its use for those who had not.

All respondents were asked to identify their role at the FHT and whether they had used the PAAF in any encounters with a patient with asthma. If they had answered “no” to the use of PAAF, they were asked to select any barriers they encountered to using the PAAF, such as their knowledge of the form and the ease of finding it in the EMR, time constraints during patient visits, perceived benefit, and a text field to identify any additional barriers. If respondents answered “yes” to using the form during encounters with a patient with asthma, the survey directed participants to the second section. In this section, they were asked to rate their agreement with various statements about the PAAF in the primary care setting on a 5-point Likert scale, such as statements commenting on the benefit of using the e-form during a visit. They were also asked to estimate the time it took to complete the form, identify barriers to its use, and provide comments on their overall experience with the form. Incomplete survey forms were excluded from analysis. The complete list of questions can be found in Multimedia Appendix 1.

Descriptive statistics were performed to analyze the survey data. Main themes were identified from the questionnaire survey results (including Likert scale responses, barriers and enablers, and free-text responses) using thematic analysis, inspired by the methodology of Braun and Clarke [30,31] to identify and summarize emerging themes from the survey responses.

## Results

### Survey Response

**Figure 1.**
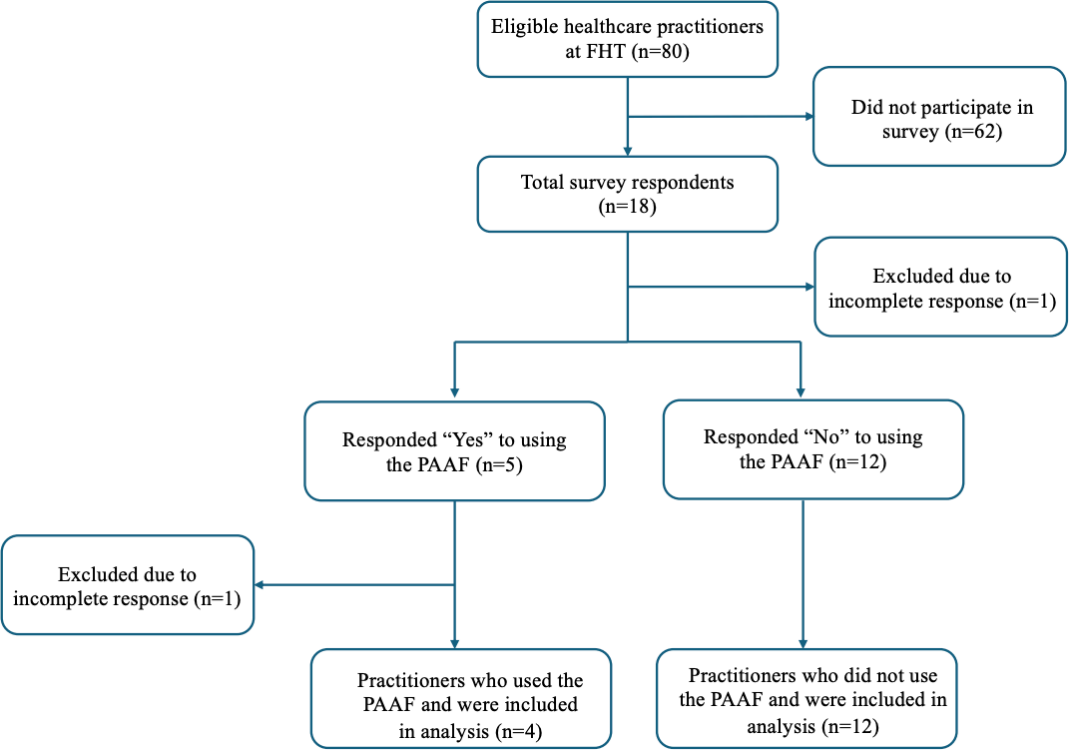
Analysis of survey respondents. FHT: family health team; PAAF: Provider Asthma Assessment Form.

**Figure 2.**
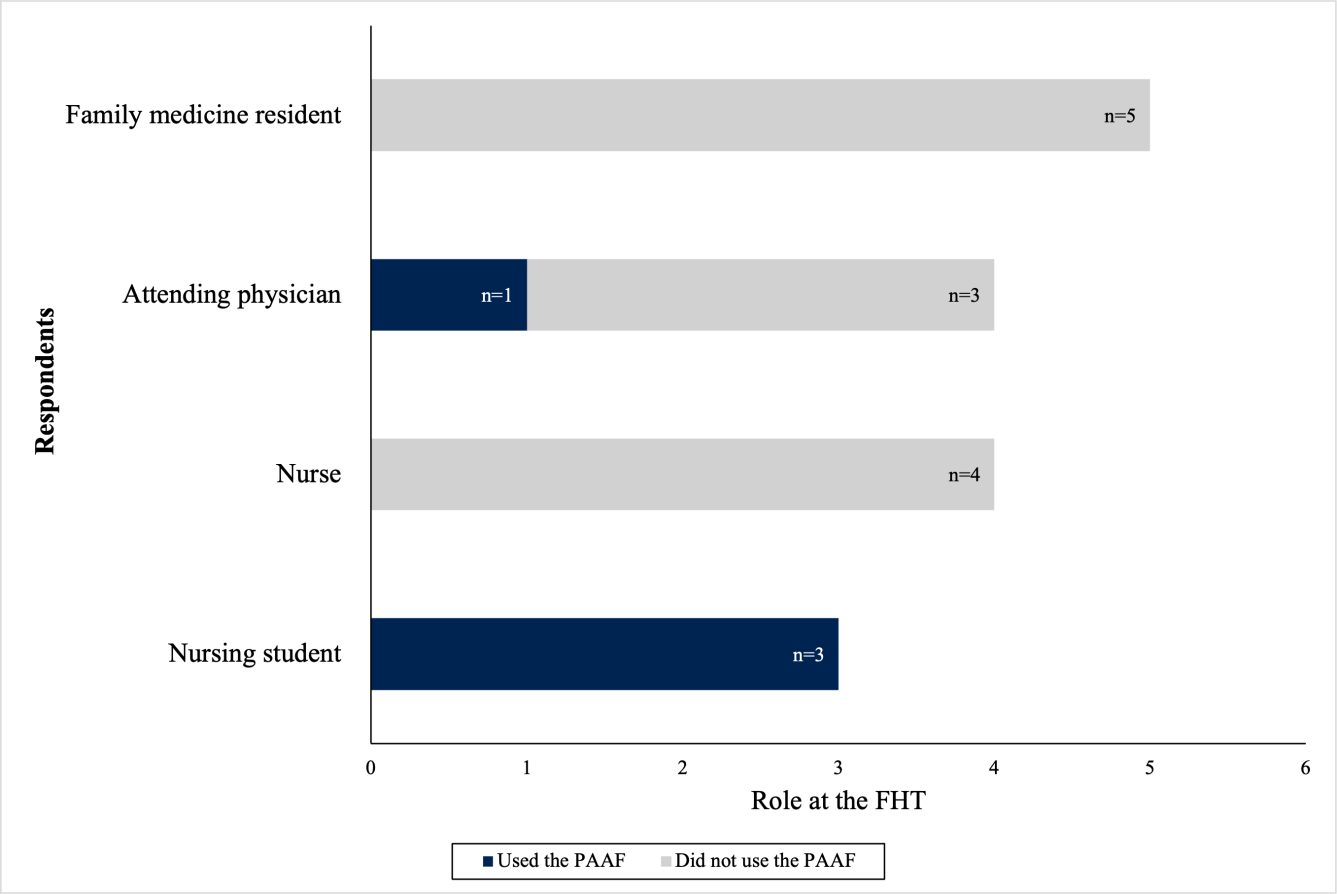
Survey respondents’ role at the family health team (FHT). PAAF: Provider Asthma Assessment Form.

Of the total 80 practitioners, 18 (22.5%) responses were recorded from the FHT practitioners (Figure 1). Of the 18 respondents, 1 (5.6%) was excluded because of an incomplete survey response. Responses were then sorted based on whether respondents had used the PAAF during an encounter with a patient with asthma. In total, 5 (27.8%) practitioners had used the form in the clinic, and 12 (66.7%) practitioners had not. Of the respondents identified as having used the PAAF, 1 (5.6%) had to be excluded due to incomplete responses to the survey questions. Respondents’ roles in the FHT are summarized in Figure 2. In total, 3 (16.7%) nursing students and 4 (22.2%) nurses responded. All 3 (16.7%) nursing students had used the PAAF during an encounter with a patient with asthma, while the nurses had not. In total, 4 (22.2%) attending physicians and 5 (27.8%) family medicine residents responded. Only 1 (5.6%) attending physician had used the PAAF, while the remaining 3 (16.7%) attending physicians and 5 (27.8%) family medicine residents had not used the form.

### Perceived Utility and Practitioner Satisfaction

**Figure 3.**
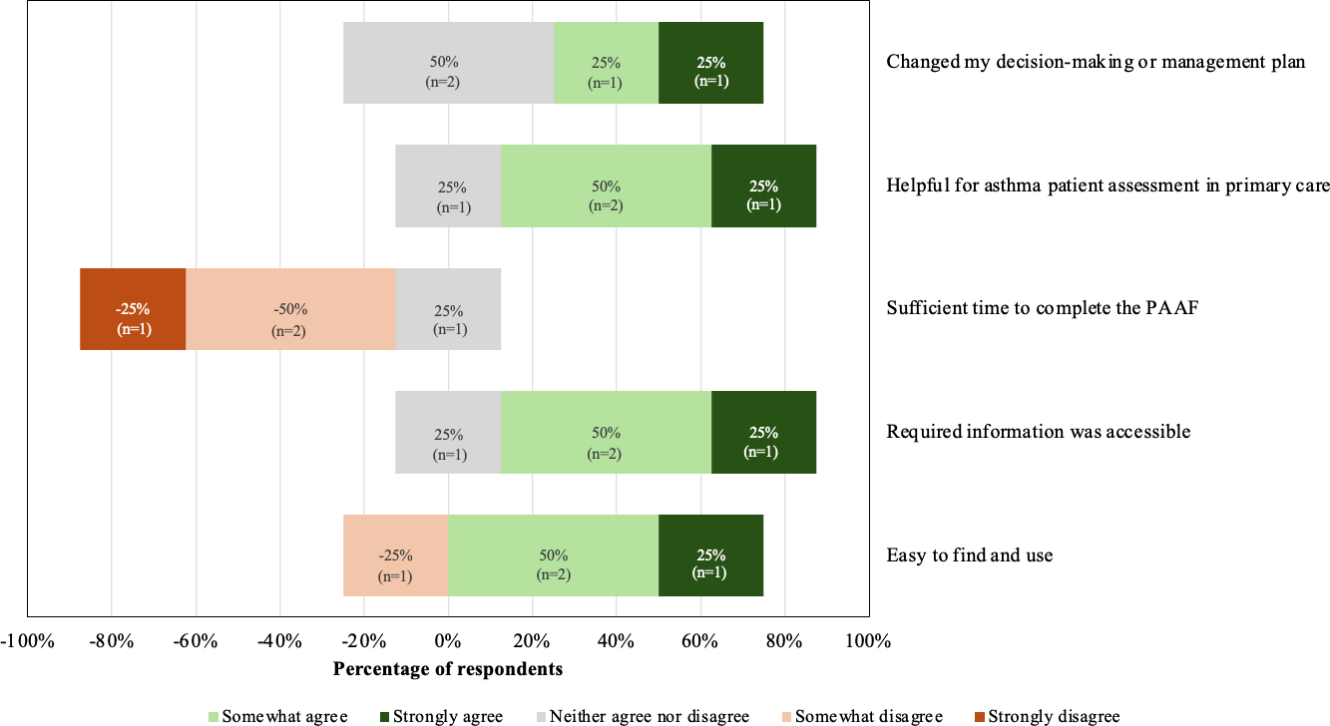
Practitioner satisfaction and perceived utility of the Provider Asthma Assessment Form (PAAF).

A 5-point Likert scale was used to assess the perceived utility and practitioner satisfaction of the respondents who used the PAAF (4/18, 22.2%; Figure 3). Respondents answered either neutral (n=2, 50%) or positively (n=2, 50%) regarding whether using the PAAF changed their decision-making and/or management plan for their patients. Most respondents (n=3, 75%) were satisfied with the PAAF as a helpful tool for assessment of patients with asthma in primary care. Most respondents were dissatisfied with the length of time it took to complete the PAAF during a standard FHT appointment (n=3, 75%). The majority were satisfied that the information required to complete the PAAF was accessible through the patient chart and/or patient history (n=3, 75%). The majority responded positively that the PAAF was easy to find and use (n=3, 75%), whereas a minority (n=1, 25%) were dissatisfied with the ease of finding and using the form.

### Identifying Barriers to PAAF Use

**Figure 4.**
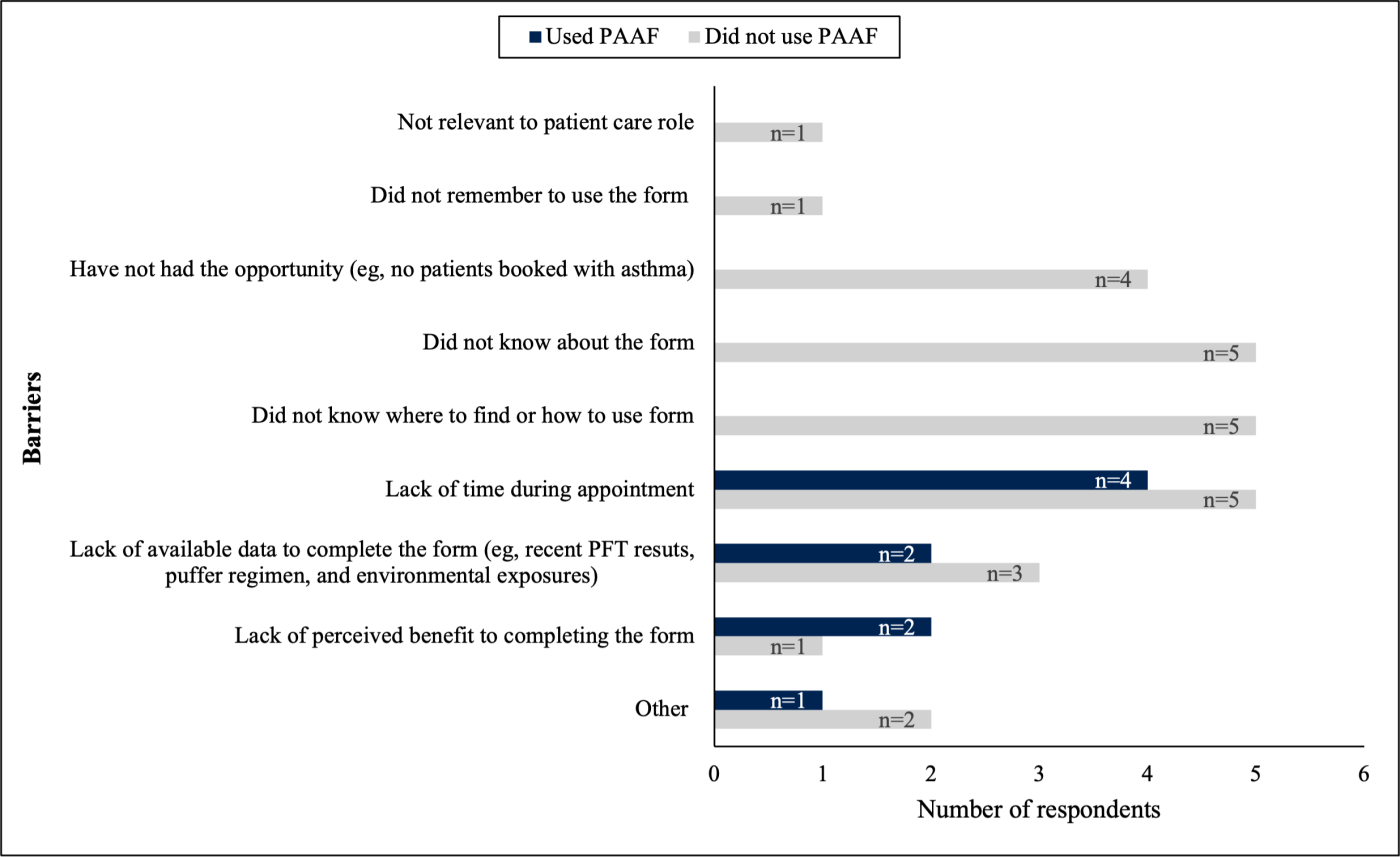
Barriers to use of the Provider Asthma Assessment Form (PAAF) at a family health team. PFT: pulmonary function test.

Respondents were surveyed regarding barriers to the use of the PAAF (Figure 4). The main barrier identified among both respondents who used the PAAF and those who did not was a lack of time during the patient encounter. Practitioners who did not use the PAAF also identified barriers such as not knowing about the form, how to use it, or where to find it; lack of available data to complete the form (ie, data from recent pulmonary function tests [PFTs], asthma medications, and environmental exposures); forgetting to use the form; perceiving it as not relevant to their patient care role; and not having the opportunity to complete the form. A minority of respondents in both groups (those who used vs those who did not use the PAAF) identified a lack of perceived benefit from completion of the form as a barrier.

The average time to complete the PAAF can be found in Table 1.

**Table 1.** Average time to complete the Provider Asthma Assessment Form (PAAF).

Time to complete the PAAF (minutes)	Responding practitioners (n=4), n (%)
0-10	0 (0)
10-20	3 (75)
20-30	1 (25)

### Perceived Benefits of the PAAF

One perceived benefit that emerged was the thoroughness of the form and the utility of the severe asthma algorithm. One respondent remarked the following:

The PAAF is a good tool to identify the current respiratory status of the patient, and the algorithm is helpful to suggest the next steps.

[Nursing student]

Several respondents who used the PAAF reported that the ability to document detailed patient respiratory history and asthma-specific data elements was beneficial (n=3, 75%).

Another respondent commented on the perceived benefit of the PAAF if some of the data elements were completed before an appointment with a patient with asthma:

The form is great, it’s just that it is hard to find the time to complete...It would be great if an allied healthcare person could pre-populate.

[Attending family physician]

### Limitations of the PAAF

A common limitation that was noted among respondents who both used and did not use the form was the length of time the PAAF took to complete. Practitioners commented that it is a good concept, but there is limited ability to complete a form with a high level of detail during a standard appointment at their primary care practice. They also expressed that completion of the form may increase workload outside of dedicated clinic hours. One respondent wrote the following:

The form has too many objective data points and is very tedious to fill out. It is a good concept, but [it is] not practical to fill out in real life with a patient and limited time during an appointment (and also outside of clinic time).

[Family medicine resident physician]

Another limitation that emerged was patients’ willingness to participate in the use of the form during a clinical encounter, especially in a virtual or phone-based appointment format. One respondent wrote the following:

[P]atients do not prioritize their respiratory health and therefore refuse to participate.

[Nursing student]

### Main Themes

Throughout the analysis of this survey, several main themes emerged regarding the barriers and enablers related to the use of the PAAF (Table 2). The primary enablers included detailed documentation, decision support, and multidisciplinary involvement. The main barriers included time constraints, knowledge and training, opportunity, availability of data, and motivation.

**Table 2.** Summary of main barriers and enablers related to use of the Provider Asthma Assessment Form (PAAF).

Themes	Barrier or enabler	Description
Detailed documentation	Enabler	The PAAF allows detailed and thorough documentation of the history of patients with asthma and its management.
Decision support	Enabler	The embedded decision support was helpful for asthma management when the PAAF was used.
Multidisciplinary involvement	Enabler	A multidisciplinary approach to use of the form, such as prepopulating the PAAF with relevant history before a visit by an allied health care professional, could facilitate uptake of the form.
Time constraints	Barrier	Insufficient time during a standard appointment to complete all components of the PAAF limited its uptake.
Knowledge and training	Barrier	A major perceived limitation to uptake of the form was a lack of knowledge and training on where to locate the PAAF and how to use it.
Opportunity	Barrier	A lack of opportunity to use the form, such as not having a patient booked for an asthma visit, limited the use of the PAAF in practice.
Availability of data	Barrier	Insufficient access to necessary data elements required to complete the form, such as the availability of recent pulmonary function test results, was a limitation.
Motivation	Barrier	Limited motivation to engage in the use of e-forms due to the perceived increase in workload for practitioners.

## Discussion

### Principal Findings

Our study revealed valuable insight and feedback regarding the implementation of a novel asthma e-tool in a primary care EMR. This study assessed practitioner satisfaction and perceived utility of the PAAF, in keeping with the KTA framework methodology, which emphasizes the importance of assessing barriers and facilitators, evaluating outcomes, and adapting KT tools throughout the process of implementation to sustain use. Several key barriers and enablers to use of the PAAF in a primary care setting emerged. Enablers of the PAAF included detailed documentation, decision support, and multidisciplinary involvement. Barriers included time constraints, knowledge and training, opportunity, availability of data, and motivation to use the e-tool. An understanding of responding practitioners’ perceived utility and satisfaction with the PAAF was also appreciated in this study. Despite the limited generalizability of this study due to its small sample size, the study identified areas for improvement in e-tools such as the PAAF and opportunities to integrate user preferences into e-tool implementation interventions.

A meaningful takeaway from this study is that various barriers persist in the implementation of novel e-tools integrated within EMRs such as the PAAF. Despite using numerous implementation strategies (including first-year resident training on the form, reminder emails, posters, a grand rounds presentation, and summary documents), uptake and use of the form were low. One barrier identified was the limited opportunity to use the e-form, as practitioners commented that they did not have or identify patients in need of an asthma assessment. Future approaches to address this barrier could involve using the newly validated case definition for adult asthma in primary care EMRs to identify patients in need of an asthma follow-up visit [32].

Despite our implementation efforts focused on educating practitioners about the form, a commonly reported barrier for respondents who had not used the PAAF was a lack of knowledge about the form, where to find it, and how to use it. To circumvent this barrier, additional dissemination and implementation strategies would need to be used. For example, more extensive education and training materials could be developed, such as an e-learning module [33]. Furthermore, the PAAF could be integrated within the formal family medicine resident curriculum or for continuing medical education credit rather than as supplementary optional presentations. Our study results emphasize that further studies evaluating the most effective implementation strategies when introducing new e-tools would help elucidate the most effective techniques and strategies to gain buy-in and interest from FHT practitioners.

Although the form was perceived by practitioners to be meaningful for asthma management, the most substantial barrier was insufficient time during a standard appointment. With an average of 10 to 20 minutes required to complete the form, practitioners believed the PAAF to be lengthy and tedious to fill out. This concern was common among practitioners who had used the form and those who had not. Time constraints within primary care practice are well-documented in the literature. A study evaluating the constraints of primary care practice highlighted that a lack of time in the multifaceted and complex primary care practice setting was a major limitation to the quality of care delivered [34]. This is an emerging theme within primary care practice settings, as concerns about physician burnout, administrative burden, and system-level challenges contribute to time constraints within primary care [35-37]. Another study emphasized the importance of developing e-tools for existing workflows and practice patterns [28]. Hence, one potential modification to increase user uptake could involve a condensed, user-friendly interface allowing practitioners to easily select the sections most relevant to a specific visit.

Overall, the findings from this study are comparable with the existing literature on asthma e-tool implementation. The PAAF was perceived to be a useful, mostly satisfactory tool in primary care by the practitioners who used the form. However, significant barriers persisted, limiting uptake and sustained use of the form in clinical practice. It is important to note that only a minority of eligible practitioners at the FHT engaged in using the form, which was a major limitation of these findings and limits the generalizability of the study results. The findings from our PAAF evaluation can be compared with those from another evaluation of primary care asthma e-tools, in which user uptake was also a limitation. The *Breathe* mobile app (for patients and practitioners) had reported high usability within primary care EMRs, but its use quickly decreased over the study duration [21,38]. Our study results, although limited by the sample size, will aid in the further development of the PAAF and future e-tools to ensure that there is a perceived benefit, real-world usability, and sustained uptake by primary care practitioners in their practice.

The results from this survey provide insight into the perceptions and beliefs of primary care practitioners when a new e-tool is introduced into their practice. In assessing the PAAF, responding providers valued the ability to document detailed disease-specific history and information, as well as the usefulness of the integrated algorithm in their clinical decision-making. Due to the high level of detail in the form, practitioners identified that a multidisciplinary approach to the e-tool’s use could increase its uptake in practice. Therefore, a valuable implementation strategy for the PAAF in its current form could involve various members of a health care team throughout the process of using it. The practitioner responses in our study highlighted the value of using a multidisciplinary approach for completing the PAAF. It may prove beneficial, for example, to involve an allied health care professional to “pre-populate” key elements of the form before the patient encounter by reviewing the available medical history and relevant data (such as recent PFT results and emergency room visits) from the patient’s chart prior to an appointment. Additionally, the PAAF could be used in an interdisciplinary asthma management program for primary care, such as the asthma-specific clinic days investigated by Licskai et al [14]. Using a multidisciplinary strategy may allow the relevant history to be completed in advance, ensuring that physicians have sufficient time during a primary care visit to complete visit-specific aspects of the form, such as asthma control; asthma severity; and care, management, and referrals.

This study was able to provide insight into perceived utility, practitioner satisfaction, and barriers and enablers related to PAAF use through a voluntary questionnaire. A strength of the study was the inclusion of both practitioners who had used the form and those who had not, providing a broader understanding of the barriers to PAAF use. Additionally, the survey included a range of health care practitioners at the FHT, including attending physicians, resident physicians, nurses, and nursing students. The inclusion of both quantitative and qualitative data elements also allowed a more diversified interpretation of the results.

It is important to recognize the limitations of this study. In this study, a mixed methods approach incorporating quantitative and qualitative data elements was used. Overall, the main limitation of this study is the lack of generalizability due to the small sample size of practitioners, with only 22.5% of FHT practitioners responding to the survey. Nonresponse bias increases the risk of the survey not being representative of all practitioners at the FHT and their barriers to using the PAAF. There is also a likelihood of selection bias, as practitioners who used the PAAF were more likely to respond to the survey. Of note, the main practitioners targeted for this study were family medicine residents since they are the group most likely to engage in asthma management at this FHT. However, all practitioners were educated on the use of the PAAF and made aware of its availability throughout the study duration. Despite the higher response rate of medical residents for the survey, they did not engage with the PAAF. It is important to recognize the known documentation and administrative burdens currently placed on primary care physicians relating to EMRs and e-tools [28,35,39]. For some practitioners, their capacity for change and the demands on their time may already be at a maximum, decreasing the motivation to try the PAAF in practice and limiting the study results.

This study would be strengthened by a larger sample size and by using different methods to engage survey nonresponders. The study may also be strengthened by incorporating patients’ perspectives when the PAAF is used during a clinic appointment. The study would also benefit from a more rigorous qualitative study design and a more in-depth follow-up study using other qualitative methodologies to fully understand practitioner perceptions of the PAAF and its use in a primary care setting.

Future directions for this work should focus on increasing the sustained use of the PAAF by adapting the e-form to address identified barriers, optimizing dissemination and implementation strategies, and evaluating widespread implementation in various primary care practice settings. Moreover, sharing the findings relating to the impact of the PAAF in primary care practice, which was the focus of another study [17], may increase motivation to integrate the form into clinical practice. Additionally, as EMRs and technology progress, it will be important to integrate user preferences into the design of e-tools to maximize their impact. For example, artificial intelligence (AI) and machine learning technology are helping to decrease the documentation burden among physicians [40]. Technology is evolving rapidly to potentially replace human chart review of medical records (allowing relevant medical history to be identified quickly within EMRs), addressing the barrier of availability of relevant data needed to complete the PAAF [41]. Furthermore, AI scribe technology may make forms such as the PAAF easier and faster to use. The integration of AI scribes within patient charts is swiftly developing, allowing the ability to autocomplete e-forms and automatically order necessary tests (ie, PFTs) during a patient encounter. Automated completion of the PAAF may aid in addressing the time constraints and documentation burdens faced during primary care patient encounters, increasing its value within primary care workflows.

### Conclusions

In this study, we gained a preliminary understanding of the perceived utility, practitioner satisfaction, and barriers and enablers associated with implementing the novel PAAF in a primary care practice. The PAAF was perceived to be a highly useful e-tool to support asthma management in primary care by survey respondents. The main barriers to uptake included time constraints, lack of awareness of the form, missing data, and lack of motivation to complete it. Future directions should include tailoring the PAAF to address these barriers and integrating the preferences of primary care practitioners. Potential strategies include multidisciplinary approaches, asthma-specific clinic days, and using forthcoming technological advancements within EMRs. Lessons learned from this study’s findings may guide future e-tool development, dissemination, and implementation within primary care EMRs.

## Supplementary material

10.2196/80399 Multimedia Appendix 1 Practitioner Asthma Assessment Form (PAAF) survey questions.

## Abbreviations

AI: artificial intelligence
CTS: Canadian Thoracic Society
EMR: electronic medical record
FHT: family health team
KT: knowledge translation
KTA: knowledge-to-action
PAAF: Provider Asthma Assessment Form
PFT: pulmonary function test
