# Supplementary material for: Implementation of an e-Tool (the Provider Asthma Assessment Form) Integrated Into the Electronic Medical Record in Primary Care: Mixed Methods Survey of Perceived Utility, Practitioner Satisfaction, Barriers and Enablers
Source: JMIR Form Res. 2026 Jun 3;10:e80399. doi: 10.2196/80399 (PMC13232603; doi:10.2196/80399)
Supplement: Multimedia Appendix 1 [file formative-v10-e80399-s001.pdf]

PAAF Survey Questions

Eligibility criteria: You are eligible to participate in this study if you are a healthcare worker providing patient care in the FHT clinic.

Survey Questions:

Section 1:

1. Please indicate your role at the Family Health Team:
    a. Family medicine resident
    b. Attending physician
    c. Nurse Practitioner
    d. Nurse
    e. Nursing student
    f. Other (please specify): _____

2. Have you used the PAAF form during asthma patient encounters? If yes, proceed to section 2. If no, please proceed to question 2.
    a. Yes
    b. No

3. If no, what barriers have you encountered to using the PAAF form? Select all that may apply.
    a. Have not had the opportunity (i.e. no patients booked with asthma)
    b. Did not know about the form
    c. Did not know where to find/how to use form
    d. Lack of time during appointment
    e. Lack of available information to complete (e.g. date of recent PFTs, puffer regimen, exposures, etc.)
    f. Lack of perceived benefit to completion of form
    g. Other: ____

Section 2: If you replied no to question 1, please answer question 2 (above) and then proceed to end of form. If you replied yes to question 1, please answer the following questions indicating how much you agree or disagree with the following statements:

1. "The PAAF form was easy to find in OSCAR and easy to use"
    a. Strongly disagree
    b. Disagree
    c. Neither agree nor disagree
    d. Agree
    e. Strongly agree
2. "The information required for the PAAF form was accessible to me through the patient chart and/or through patient history"

a. Strongly disagree
b. Disagree
c. Neither agree nor disagree
d. Agree
e. Strongly agree
f.

3. “There was sufficient time to complete the PAAF form during a standard QFHT appointment”
    a. Strongly disagree
    b. Disagree
    c. Neither agree nor disagree
    d. Agree
    e. Strongly agree

4. “The PAAF form is helpful to assess asthma patients in primary care”
    a. Strongly disagree
    b. Disagree
    c. Neither agree nor disagree
    d. Agree
    e. Strongly agree

5. “Using the PAAF form changed my decision-making and/or management plan”
    a. Strongly disagree
    b. Disagree
    c. Neither agree nor disagree
    d. Agree
    e. Strongly agree

Please answer the following questions:

1. How long did the completion of the PAAF form take?
    a. <10 minutes
    b. 10-20 minutes
    c. 20-30 minutes
    d. >30 minutes

2. What barriers have you encountered to using the PAAF form? Select all that apply)
    a. Difficulty finding and/or using the form
    b. Lack of time during appointment
    c. Lack of available information to complete (e.g. date of recent PFTs, puffer regiment, exposures, etc.)
    d. Lack of perceived benefit from completion of form
    e. Other: ______

f. No barriers

3. Please provide any comments regarding benefits or positive experiences with the PAAF form:
    a. Open box for comments

4. Please provide any comments regarding barriers or negative experiences with the PAAF form:
    a. Open box for comments
